# Community-based behavioural activation for depression in adolescents: feasibility study, survey and stakeholder consultations

**DOI:** 10.3389/frcha.2025.1596294

**Published:** 2025-07-04

**Authors:** Lucy Tindall, Emily Hayward, Jinshuo Li, Philip Kerrigan, Susan Metcalfe, Lina Gega

**Affiliations:** ^1^ Department of Health Sciences, University of York, York, United Kingdom; ^2^ Research and Development, Tees Esk and Wear Valleys NHS Foundation Trust, Durham, United Kingdom; ^3^ Hull York Medical School, University of York, York, United Kingdom

**Keywords:** low mood, young people, psychological therapy, school interventions, usual care

## Abstract

**Background:**

Behavioural activation, a brief psychological therapy for depression across the lifespan lends itself well for delivery in community settings (e.g., non-hospital health services, schools, charities). Ahead of a randomised controlled trial, we wanted to “road-test” our recruitment and assessment processes, intervention materials and data collection tools, and understand (1): how BA can be delivered in community settings and by whom, (2) whether young people will adopt and complete it, (3) whether there are any observed changes in depression and anxiety and (4) whether usual care would be a feasible comparator.

**Methods:**

In three settings—one community-based child and adolescent mental health service, one school, one charity—we offered up to 8 sessions of behavioural activation to 12–18-year-olds with mild-to-moderate depression. Stakeholder consultations helped us develop our research materials and processes. Self-report questionnaires assessing depression, anxiety, quality-of-life and resource use were completed by participants at baseline and 8-weeks. Professionals completed an online questionnaire about usual care for young people with depression in different settings, including types of support and staff delivering it.

**Results:**

Twenty young people (average age 15 years, 17 females) consented; of those, 19 attended behavioural activation sessions (*M* = 7.4, *SD*: 1.5) and all 20 completed baseline and follow-up measures. For three-quarters of participants there was a “positive” change in scores (defined as a drop of ≥1 on the RCADS) from baseline to follow-up across all measures. A Resource Use Questionnaire for Adolescents collecting information about use of hospital and community-based health and social care services was developed and tested during the study. Intervention costs were modest at £207 (*SD*: £79) per participant for just over 5 h (*M* = 286 min, *SD* = 63 min) of contact on average with a professional.

**Conclusions:**

Excellent intervention uptake and adherence (implying robust recruitment and assessment processes), retention to follow-up and data completeness, and a positive direction of change across all outcome measures justify the need for a fully powered randomised controlled trial comparing community-based behavioural activation with usual care for adolescents with mild-to-moderate depression. Furthermore, usual care rarely included behavioural activation, which made it a suitable comparator for a future randomised controlled trial.

**Trial Registration:**

https://doi.org/10.1186/ISRCTN30483950, identifier (ISRCTN, ISRCTN304839502).

## 1 Introduction

Depression is currently the fourth leading cause of illness and disability among adolescents aged 15–19 and fifteenth for those aged 10–14 (1). In the UK, several evidence-based psychological interventions for young people experiencing low mood/depression are recommended by the National Institute for Health and Care Excellence (2). These are routinely delivered within Child and Adolescent Mental Health services (CAMHS) and include watchful waiting, medication, Cognitive Behavioural Therapy (CBT), Interpersonal Therapy (IPT) and Non-Directive Supportive Therapy (NDST).

Adolescence is an important developmental period and experiencing depression during this time can have a negative impact both in the short-term and spanning into adulthood (3). It is therefore essential that young people receive effective treatments at the earliest opportunity. However, owing to increasing demand and limited resources in CAMHS there is an evident gap between treatment provision and need with many services having long waiting lists and high entry thresholds (4). As a result, many young people with depression are not able to access timely clinical interventions. In response, more emphasis has been placed on expanding therapy provision into non-NHS services, including schools (5). It is therefore timely to examine treatment options that may be effectively delivered more broadly outside NHS services.

One treatment that can be delivered within various settings and by professionals with different levels of expertise (6), including non-specialists outside clinical services (7–9), is Behavioural Activation (BA); however, most evidence comes from adult populations with depression [e.g., (7–9)], or within specialist services for children and adolescents (10).

BA is a depression-specific brief psychological intervention with the fundamental aim of restoring and increasing stable sources of positive reinforcement in a person's environment. This is achieved through planning and engaging in purposeful and rewarding activities that have a positive emotional impact on an individual's mood, interest in people and life, and energy levels (11). BA requires fewer sessions (12) and shorter training than several more established therapies (e.g., CBT, IPT), making it a promising less-resource intensive alternative. Furthermore, BA's focus on withdrawal, inactivity, and avoidance, which are common symptoms of depression in young people, may make it better suited for this group (13).

BA is a recommended intervention for adults experiencing depression (14), but it does not currently feature in any national (2) or international (15) recommendations for young people. Previous reviews (16, 17) supported BA as a promising intervention for depression in adolescents. We conducted a recent systematic review (18) in which we identified 23 papers (6 RCTs and 17 pre-post evaluations) relevant to BA with children and adolescents. Our meta-analysis found a small effect of 0.24 in favour of BA compared to a waiting list control, usual care and other therapies. There was not enough evidence about the effects of BA and value for money. Out of the included studies 8 were conducted face-to-face in the community including within schools. A wide range of professionals (e.g., students, social workers, school counsellors) were able to successfully deliver BA within these studies. This provides evidence to support the expansion of evidence-based mental health interventions, like BA, for young people beyond health services. However, the maximum sample recruited across studies was 35 and those that were UK-based (*n* = 2) only had a collective sample of 10 participants. These findings support the need to conduct a fully powered trial, including within UK community-settings, and this feasibility study will inform this.

We want to evaluate the potential of delivering BA in community settings before young people enter specialist psychiatric services. To this end, we developed a BA manual and associated training materials for use within community settings such as schools and charities. Having reviewed the manuals identified within our systematic review, we concluded that there was no single manual that included all the necessary components for our target audience. Specifically, no one manual was developed for use with adolescents (aged 12 to 18 years) experiencing mild-to-moderate low mood/depression within community-based settings in the UK. Furthermore, where others produced different versions for young people, parents/guardians and professionals we wanted a single manual useable by all.

As a precursor to a randomised controlled trial (RCT), we wanted to understand: how BA can be delivered in community settings and by whom, whether young people will take it up and complete it, and whether usual care would be a feasible comparator. To enable the capture of the impact of BA delivery on wider services use in a RCT, we also wanted to explore and identify what other healthcare and social services were currently accessed in this population and how frequently they were accessed, given the ever-changing provision and less than structured community services across areas.

The aims of this study were to: (a) evaluate the feasibility and acceptability of delivering BA in community settings, (b) develop and road-test bespoke intervention materials and data collection tools in consultation with young people, parents and professionals, and (c) rehearse the research processes, in preparation for a fully powered RCT comparing BA's clinical and cost effectiveness against usual care. The study had the following objectives:

1. Measure intervention uptake, adherence, completion of follow-up measures and data missingness in the questionnaires.
2. Observe the direction of change in depression and anxiety when young people use our newly developed BA materials, with support from professionals.
3. Develop a Resource Utilisation Questionnaire for Adolescents (RUQ-A) to explore and identify the main service use associated with depression for NHS and non-NHS organisations accessed by participants.
4. Map what “usual care” means across the range of community settings where young people may seek support for depression.

## 2 Materials and methods

### 2.1 Design, settings and participants

This pre-post single group feasibility study, with an embedded survey and a series of stakeholder consultations, was conducted between March and July 2022. Participants were recruited from three services: one child and adolescent mental health service, one school and one charity, in the North of England. Our eligible population was young people aged 12 to 18 years with mild-to-moderate depression. Eligibility was defined by a T-score of ≥65 on the depression subscale (10-items) of the Brief Revised Children's Anxiety and Depression Scale [RCADS-25 (19)]. Young people for whom secondary/specialist care was a more appropriate option (e.g., due to risk of suicide, severe depression, learning disability) did not participate in the study but were signposted to appropriate local services by the research team.

### 2.2 Procedure

Within recruiting sites, professionals provided potential participants with study information sheets, an expression of interest form, and the RCADS-25 depression subscale. Those interested returned their completed expression of interest form and RCADS-25 questionnaire to the research team who confirmed eligibility. A baseline meeting (either face-to-face or online) was arranged with those eligible. Here, fully informed written assent/consent was obtained (alongside parental consent if a young person was ≤15 years old), and young people were asked for some basic demographic information (i.e., age, sex, ethnicity, religion, family circumstances and education or work).

### 2.3 Outcome measures

All young person participants completed the following self-report questionnaires with a researcher at week 0 (baseline) and at 8 weeks post-baseline (follow-up).

*Revised Children's Anxiety and Depression Scale*—25 item [RCADS-25 (19)]: A condensed version of the original 47-item (20), the RCADS-25 assesses depression and anxiety and has been validated as a self-completed measure for 8–18-year-olds. The RCADS-25 has subscales that capture symptoms across six domains: one domain is depression (10 items) and five domains relate to different anxiety problems (15 items). All items are rated on a 4-point Likert-scale from 0 to 3, where 0 = Never, 1 = Sometimes, 2 = Often, and 3 = Always. Raw scores are transformed into T-scores with higher T-scores denoting greater clinical need. We report T-scores for the RCADS-25 with clinical cut-offs of: 0–64 non-clinical range, 65–69 sub-clinical range, and ≥70 clinical range. The clinical cut-offs are the same for the depression and anxiety subscales (21).

The RCADS-25 was our primary outcome measure and was selected because it is a nationally recommended outcome measure as part of the Mental Health Services Dataset (MHSDS) in the UK. It is routinely collected by professionals within clinical services with children and young people and it is used by commissioners for service evaluation and benchmarking. Therefore, our findings can be interpreted in the context of UK national datasets for our population.

*Children's Depression Rating Scale-Revised* [CDRS-R (22)]: A 17-item researcher-administered interview widely used in clinical research as a validated measure to assess severity of, and change in, depression symptoms in children and adolescents (23). The CDRS-R covers seventeen symptom areas (e.g., dysfunction, interpersonal relationships, psychosomatic complaints) with items scored on a 1–5 or 1–7 scale. We report raw scores which range from 17 to 113, with higher scores denoting more depressive symptoms. Scores ≤34 suggest no depression, a score between 35 and 40 suggests emerging or early depression (24) and scores >40 indicate diagnosable depression (23).

*Behavioural Activation for Depression Scale- Short Form* [BADS-SF (25)]: A 9-item questionnaire, based upon the longer 25-item BADS (26, 27), that measures levels of activity on 2 subscales: activation and avoidance. All items, based on the previous week, are rated on a seven-point scale ranging from 0 (not at all) to 6 (completely) with total scores between 0 and 54; higher scores represent increased behavioural activation. Although not currently validated with adolescents, we used the BADS-SF as there are no alternative similar tools available.

*Child Health Utility-9 Dimensions* [CHU-9D (28)]: The CHU-9D is a self-complete paediatric generic preference-based measure validated for young people aged 7–17 years (29). It was used in the place of the conventional EQ-5D to derive utility values for estimating quality-adjusted life years (QALYs). This was because the youth version of EQ-5D does not have a validated UK tariff and the adult version is not validated in adolescents. The questionnaire assesses functioning “today” across 9 domains (worry, sadness, pain, tiredness, annoyance, school, sleep, daily routine, and activities), each of which has 5 levels (scored 1–5, 1 as best, 5 as worst). Each level has a tariff value smaller than 1 estimated based on population survey (30). For example: in the sadness domain, 1 = I don’t feel sad today (0), 2 = I feel a little bit sad today (0.420), 3 = I feel a bit sad today (0.0455), 4 = I feel quite sad today (0.0722), 5 = I feel very sad today (0.0722). Once all 9 domains are complete, the tariff values of selected levels of them are extracted from 1 (a full utility). The resulting utility value ranges from 0.3261–1, where 1 is perfect health and death is anchored on 0. Utility values at multiple time points are used to estimate QALYs using area under the curve approach (31) and assuming linear changes between time points.

*Resource Utilisation Questionnaire for Adolescents (RUQ-A):* The RUQ-A was developed by us specifically for this study to collect quantities of use of primary (e.g., GP, practice nurse, etc.), secondary (e.g., A&E, inpatient, outpatient, etc.), community- (e.g., CAMHS team, community paediatrician, social worker, etc.) and school-based (e.g., school nurse, teacher, etc.) healthcare and social services by participants over the preceding 8 weeks via self-report. The scope was based on the reference case for a formal economic evaluation by NICE (32). The initial version collected the use of these services separately for mental health and physical health. Following its completion with the first four participants, we made two amendments to the RUQ-A based on their feedback: merged questions about mental and physical wellbeing, i.e., not differentiating between the two, and adding a question about medication. The amended version included 4 questions for healthcare and social services use and one for days absent from school, training or work. Participants were asked to report the number of times they consulted with the listed professional roles or services in primary and community settings (including at school, in CAMHS, at hospital and private settings) and the respective number of times, average duration, and any out-of-pocket payments (for private settings only). Free text was provided for each question if the service they used or professional consulted with was not on the list. The last question asked participants the number of days they missed from school, training or work as well as the number of days their carers took off work to care for them. The listed items in questions were informed by a literature search of questionnaires previously used in economic evaluations in this population and by previous RUQs developed by our team for mental health interventions. The results were used to identify the frequently accessed services so the collection of this data could be streamlined in the full RCT.

### 2.4 Intervention content

Our BA manual was designed for use by young people and their supporting professionals and parents/guardians and was informed by McCauley et al's (6) clinician's guide among other available BA resources. We have singled out McCauley et al's (6) guide because its application has the best published evidence in terms of the feasibility, acceptability and clinical outcomes of BA for young people to-date. We have developed our BA manual “*de novo*”. This was for three main reasons: (a) it focuses on adolescents with mild-to-moderate presentations of depression, (b) it can be delivered by non-specialists in community settings (c) it is a single point of reference for young people, parents/guardians and professionals (rather than having different versions of it for each audience).

The manual was divided into 5 modules: Module 1-Starting Up, Module 2-Getting Active, Module 3-Building Skills, Module 4-Overcoming Obstacles and Module 5-Moving Forward. The manual was not a self-help book, but the young person worked through the modules with support from a professional. Each module could be completed in one session, or it could be done in two or more sessions. The modules had two key components: “Things to Know”—information about key concepts and strategies of Behavioural activation (BA)—and “Things to Do”—practical activities that could take place either in-session with the professional or between sessions as “take-away activities”.

### 2.5 Intervention delivery

BA sessions were delivered face-to-face or online via a secure NHS platform. All participants were offered up to 8 weekly sessions of BA, each lasting between 30 and 45 min. Each session followed the manual for both in-session and between-session work. The young person could attend these sessions with a parent/guardian or another trusted adult who could offer them practical and emotional support outside the sessions.

Professionals were offered weekly supervision in a flexible format: one-to-one or in a group, in person or online. All professionals received a full day's training either in person or online via Microsoft Teams, in addition to completing self-directed learning through an online Massive Online Open Course [MOOC (33)].

Professionals were asked to keep a written record of all BA sessions, noting whether the session took place or was missed, how long it lasted, what activities were completed, whether there were any adverse/serious adverse events reported during the session. The professionals also tracked between-session communications with young people or their parents (e.g., why and how they made contact and the outcome of the communication).

### 2.6 Sample size

There is limited guidance about the optimum sample size for feasibility studies (34). Julious (35) recommends, as a rule of thumb, recruiting a minimum of *n* = 12 for a single-group feasibility study. We aimed to recruit *n* = 15 young people, slightly inflating the number recommended by Julious (35) to account for possible attrition.

### 2.7 Data analysis

We reported the number of people who took up the invitation and consented to participate in the study, the number of participants who completed the intervention or withdrew, and the number of those who completed follow-up measures. We also reported data missingness in the questionnaires. We calculated three sets of T-scores for each participant on our primary outcome measure (RCADS-25): the overall score and the depression and anxiety subscale scores. Using established clinical cut-offs for the RCADS-25 scale, we assigned each participant to one of three groups based on their T-score: A = non-clinical (scores 0–64), B = sub-clinical (scores 65–70) and C = clinical (scores 70+) (21). For the CDRS-R, we reported both the continuous raw scores and the group to which each participant belonged to, based on their scores using the CDRS-R cut-offs: A ≤ 34 = no depression, B = 35–40 emerging/early depression, C > 40 = diagnosable depression.

We created scatterplots to visually present the direction of change in the outcome measures based on the difference in scores from baseline to follow-up. We created bubble-plots to visually present the transition of participants from one group to another (e.g., from clinical to sub-clinical depression). We calculated the means and standard deviations of the RCADS-25 T-scores and CDRS-R raw scores in our sample as an indication of the average severity of depression at baseline and at follow-up. As this was a feasibility study, we did not conduct statistical analyses to measure effects, but we used the term “direction of change” as an indication of the potential effect of the intervention that could justify a follow-up RCT. The terms “positive” and “negative” direction of change do not imply statistically or clinically significant change.

### 2.8 Cost analysis

Using research team records, the operating costs of the BA were calculated in Pound Sterling 2022 price and presented by cost elements. RUQ-A data were used to identify the main services associated with depression for NHS and non-NHS organisations that were accessed by participants. The results, alongside participant feedback, were used to inform the revision of the RUQ-A for use in the future RCT. The CHU-9D was used to measure health-related quality of life but given the short observation period, quality-adjusted life years were not calculated.

### 2.9 Usual care survey

We created a short survey hosted on the Qualtrics Platform to gather information about the types of support available to young people with depression in different settings, the professionals responsible for providing this and to determine the extent to which BA was already being used. The survey was distributed to professionals within approximately 40 organisations in the North-East of England working with young people within specialist mental health services, mainstream and special schools, NHS Talking Therapies, voluntary agencies and youth organisations.

### 2.10 Stakeholder consultations

Ahead of the feasibility study we identified three key stakeholder groups—young people (aged 12 to 18 years as per the recruitment group) with lived experience of low mood/depression, parents/guardians of children with such lived experience, and professionals with experience of delivering low-intensity psychological interventions. The young person and parent/guardian groups were identified from several local Patient and Public Involvement and Engagement (PPIE) organisations/networks as well as our study sponsor's PPIE register. Professionals were identified from the professional networks of the study team. Our stakeholder consultations were critical to the development of the format and content of the BA as well as informing the design of key documentation for the feasibility study.

We completed two rounds of consultation with our stakeholders, either online or in person. All consultations included an initial presentation on the core intervention and study materials which were then discussed as a group. The first round included workshops with the three stakeholder groups (three professionals, four young people and three parents/guardians) where we gathered feedback on drafts of the BA manual, the RUQ-A, participant information sheets and assent/consent forms for use in the feasibility study. The feedback received helped in shaping these key materials, with the subsequent changes reported back in our second round of consultations. During this second round, our three stakeholder groups also provided feedback on some newly devised content in the BA manual, plus a new question about medication added to the RUQ-A.

## 3 Results

### 3.1 Participant characteristics

Our sample included 20 young people aged 12–18 years (*M* *=* 14.9, *SD* *=* 1.6), recruited over 5 months (February-June 2022) from three sites: an NHS service, a school and a charity. They were predominantly British (i.e., identified as English, Welsh, Scottish or Northern Irish), except 4 participants who identified as: White and Asian, Pakistani, Bangladeshi and African. Just over half of the sample (*n* = 11) were not affiliated to any religion (agnostic or atheist), 6 were Christians, 2 Muslims and 1 Hindu. All young people lived with their parents, and most were female (*n* = 17). Our participants attended secondary school (*n* = 14) or were in further/higher education (*n* = 6); only 5 were concurrently working (1 full-time, 4 part-time). The baseline RCADS-25 T-scores (based on the depression sub-scale) ranged from 65 to 80, but, on average, the sample scored above the threshold for likely clinical depression (*M* *=* 72.5, *SD* *=* 5.5). All information is depicted in Supplementary Table S1.

### 3.2 Professional characteristics

Overall, five professionals delivered the BA and were based within one school, one charitable organisation, and one child and adolescent mental health service. The professionals included a school counsellor, a senior children's wellbeing practitioner, an art therapist and 2 trainee children's wellbeing practitioners. Most (*n* = 4) were female and aged between 25 and 34 years (*n* = 4).

### 3.3 Uptake, completion and follow-up

All young people who were invited to participate consented to do so and all participants completed baseline and follow-up measures. All but one participant received the intervention. The one participant who withdrew prior to receiving the intervention was at the clinician's suggestion (BA was not deemed to be the most suitable course of treatment for this young person); they still completed outcome measures at follow-up. No data was missing from any of the completed baseline and follow-up questionnaires.

Among the 19 participants who received BA, the mean number of sessions attended was 7.4 (*SD:* 1.5), ranging from 2 to 9 sessions per person and the mean duration per session was 39 min (*SD:* 4 min). Over half of the participants (*n* = 11) attended their sessions as planned without any cancellations, whereas for the rest the mean number of cancelled or “no-show” sessions was 1.2 (*SD:* 2.0). For most participants (*n* = 14) sessions took place every 7–10 days, for 1 participant, sessions occurred every 11 days on average, and for another 3 participants the frequency of sessions was more than one per week. One participant was an outlier, having had only 2 sessions over 3 months (92 days). In total, participants on average spent 286 min (*SD:* 63 min) over the course of delivery.

The time practitioners spent in direct contact with the young people during BA sessions is reported above. In addition, practitioners spent an average of 103 min (*SD* = 57 min) preparing for sessions and 130 min (*SD* = 41 min) doing administrative tasks (e.g., writing notes). For cancelled/not attended sessions, professionals still spent time on preparation (*M* = 13 min, *SD* *=* 26 min) and administration (*M* = 11 min, *SD* = 20 min). No adverse nor serious adverse events were reported during the feasibility study.

### 3.4 Change in screening for eligibility

The lack of severity cut-off scores on the RCADS 10-item depression subscale made it difficult for us to differentiate between mild-moderate and severe depression at the point of screening for eligibility to enter the study. Consequently, we added the 9-item Patient Health Questionnaire for Adolescents [PHQ-9A (36)] as a screening tool, in addition to the RCADS-25. If a young person scored ≥15 or gave answers indicating suicidal thoughts based on items of the PHQ-9A, we invited them to a clinical interview to assess depression severity and risk. If the clinical interview judged that the depression was mild-to-moderate, then the young person was invited to participate in the study; otherwise, the clinicians signposted or referred the young person to an appropriate service.

### 3.5 Direction of change in outcomes

#### 3.5.1 RCADS—25

At baseline, for the sample, the average RCADS-25 total T-score was 65.7 (*SD* = 9.8), which falls within the sub-clinical range, whereas the depression subscale T-score was 70.5 (*SD* = 7.5)—just above the clinical threshold—and the anxiety subscale T-score was 57.2 (*SD* = 10), which falls in the non-clinical range. At follow-up, all three mean RCADS-25 T-scores fell within the non-clinical range: the total mean T-score was 57 (*SD* = 10.1), the depression mean T-score was 62.2 (*SD* = 9.8) and the anxiety mean T-score 51.8 (*SD* = 10.4).

**Figure 1:**
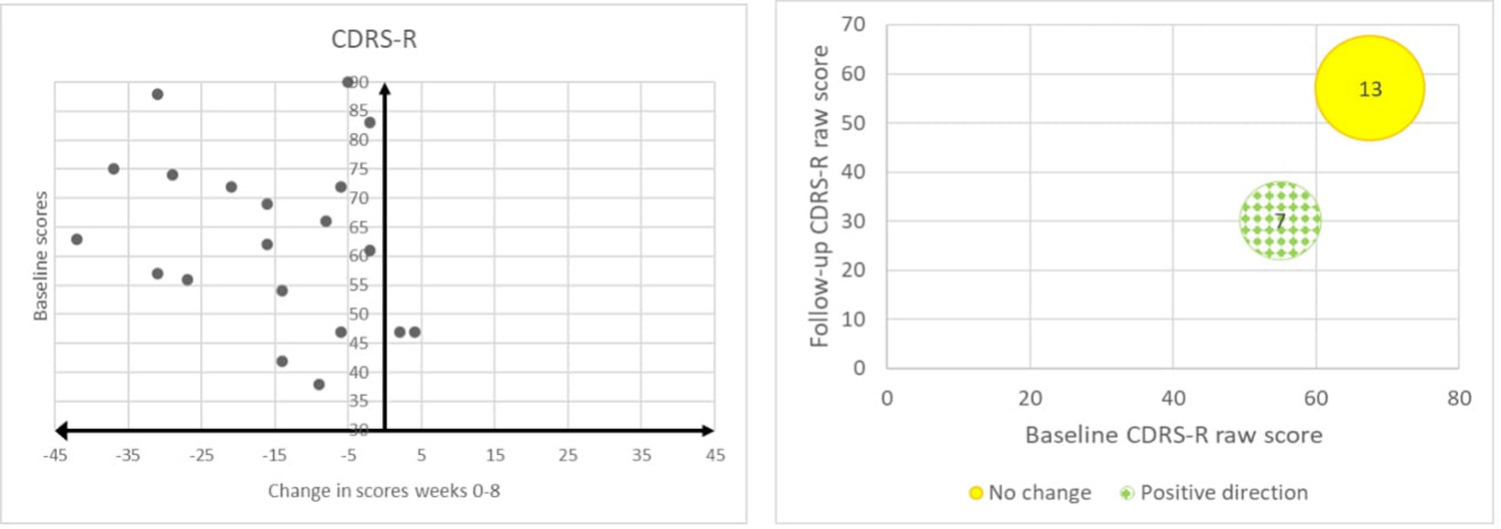
Scatterplots and bubble-plots indicating direction of change for each participant based on their RCADS scores (total, depression, anxiety) and transition in clinical status.

Tables 1–3 show the breakdown of individual participant T-scores. Within 8 weeks, scores changed in a positive direction for 16/20 participants on the RCADS-25 overall, for 15/20 participants on the depression subscale and for 14/20 participants on the anxiety sub-scale. The scatterplots Figure 1 illustrate the distribution of the changes in scores from baseline to follow-up where negative values indicate changes in a positive direction.

**Table 1:** RCADS-25 total scores at baseline (week 0) and follow-up (week 8).

ID	RCADS-25 overall scores						
	T-score		Diff.	Direction of change^a^ Weeks 8–0	Group^b^		Direction of change^c^ Weeks 8–0
	Week 8	Week 0			Week 8	Week 0	
01	50	50	0	None	A	A	None
02	69	50	−19	Negative	B	A	Negative
03	46	54	+8	Positive	A	A	None
04	53	58	+5	Positive	A	A	None
05	48	64	+16	Positive	A	A	None
06	49	76	+27	Positive	A	C	Positive
07	40	54	+14	Positive	A	A	None
08	69	74	+5	Positive	B	C	Positive
09	55	59	+4	Positive	A	A	None
10	52	69	+17	Positive	A	B	Positive
11	71	80	+9	Positive	C	C	None
12	61	80	+19	Positive	A	C	Positive
13	47	67	+20	Positive	A	B	Positive
14	56	80	+24	Positive	A	C	Positive
15	68	64	−4	Negative	B	A	Negative
16	80	73	−7	Negative	C	C	None
17	61	59	+2	Positive	A	A	None
18	59	68	+9	Positive	A	B	Positive
19	54	61	+7	Positive	A	A	None
20	52	73	+21	Positive	A	C	Positive

^a^ Direction of change based on scores at week 8 compared to baseline (week 0) is indicted as positive if scores were higher at baseline (denoting more severe depression) than at 8 weeks.

^b^ Groups based on RCADS-25 score thresholds: A = 0–64 non-clinical, B = 65–70 sub-clinical, C = 70 + clinical.

^c^ Direction of change based on clinical status at week 8 compared to baseline (week 0) is indicated as “positive” if a participant changed groups in descending alphabetical order i.e., from C to B or A.

**Table 2:** RCADS-25 depression subscale at baseline (week 0) & follow-up (week 8).

ID	RCADS-25 depression subscale						
	T-score		Diff.	Direction of change^a^ Weeks 8–0	Group^b^		Direction of change^c^ Weeks 8–0
	Week 8	Week 0			Week 8	Week 0	
01	59	66	+7	Positive	A	B	Positive
02	80	67	−13	Negative	C	B	Negative
03	51	65	+14	Positive	A	B	Positive
04	59	70	+11	Positive	A	B	Positive
05	57	57	0	None	A	A	None
06	51	76	+25	Positive	A	C	Positive
07	51	76	+25	Positive	A	C	Positive
08	59	80	+21	Positive	A	C	Positive
09	59	68	+9	Positive	A	B	Positive
10	59	80	+21	Positive	A	C	Positive
11	80	80	0	None	C	C	None
12	75	80	+5	Positive	C	C	None
13	62	67	+5	Positive	A	B	Positive
14	63	80	+17	Positive	A	C	Positive
15	70	64	−6	Negative	B	A	Negative
16	80	73	−7	Negative	C	C	None
17	54	59	+5	Positive	A	A	None
18	64	68	+4	Positive	A	B	Positive
19	51	61	+10	Positive	A	A	None
20	59	73	+14	Positive	A	C	Positive

^a^ Direction of change based on scores at week 8 compared to baseline (week 0) is indicted as positive if scores were higher at baseline (denoting more severe depression) than at 8 weeks.

^b^ Groups based on RCADS-25 score thresholds: A = 0–64 non-clinical, B = 65–70 sub-clinical, C = 70 + clinical.

^c^ Direction of change based on clinical status at week 8 compared to baseline (week 0) is indicated as “positive” if a participant changed groups in descending alphabetical order i.e., from C to B or A.

**Table 3:** RCADS-25 anxiety subscale at baseline (week 0) and follow-up (week 8).

ID	RCADS-25 anxiety subscale						
	T-score		Diff.	Direction of change^a^ Weeks 8–0	Group^b^		Direction of change^c^ Weeks 8–0
	Week 8	Week 0			Week 8	Week 0	
	44	39	−5	Negative	A	A	None
	57	66	+9	Positive	A	B	Positive
	43	45	+2	Positive	A	A	None
	48	47	−1	Negative	A	A	None
	42	66	+24	Positive	A	B	Positive
	47	70	+23	Positive	A	B	Positive
	32	48	+16	Positive	A	A	None
	74	63	−9	Negative	C	A	Negative
	52	50	−2	Negative	A	A	None
	47	55	+8	Positive	A	A	None
	62	60	−2	Negative	A	A	None
	51	63	+12	Positive	A	A	None
	38	49	+11	Positive	A	A	None
	49	68	+19	Positive	A	B	Positive
	63	70	+7	Positive	A	B	Positive
	66	68	+2	Positive	B	B	None
	64	61	+3	Positive	A	A	None
	54	42	−12	Negative	A	A	None
	56	62	+6	Positive	A	A	None
	46	51	−5	Positive	A	A	None

^a^ Direction of change based on scores at week 8 compared to baseline (week 0) is indicted as positive if scores were higher at baseline (denoting more severe depression) than at 8 weeks.

^b^ Groups based on RCADS-25 score thresholds: A = 0–64 non-clinical, B = 65–70 sub-clinical, C = 70 + clinical.

^c^ Direction of change based on clinical status at week 8 compared to baseline (week 0) is indicated as “positive” if a participant changed groups in descending alphabetical order i.e., from C to B or A.

When considering diagnostic cut-offs, the number of participants who transitioned from clinical or sub-clinical to non-clinical status were: 8/20 based on their RCADS-25 total T-scores, 12/20 on the depression subscale T-scores and 5/20 on the anxiety subscale T-scores. The remaining participants either did not change diagnostic status, or transitioned from non-clinical or sub-clinical to clinical status. The bubble-plots in Figure 1 illustrate the number of participants who changed clinical status (positive direction, negative direction or no change), relative to their baseline and follow-up T-scores.

#### 3.5.2 Children's depression rating scale-revised (CDRS-R)

**Figure 2:**
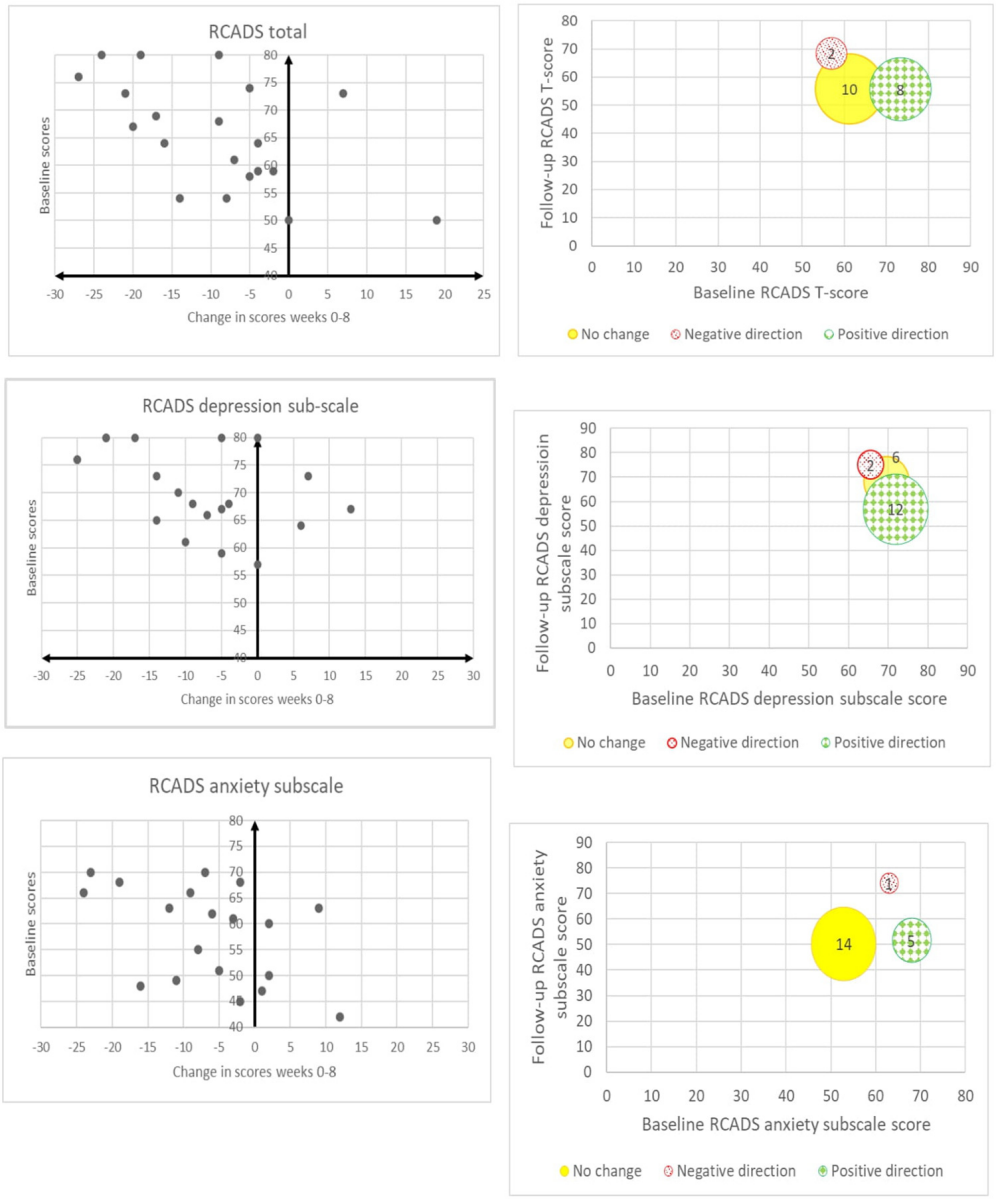
Scatterplot and bubble-plot indicating direction of change for each participant based on their CDRS-R raw scores and transition in clinical status.

For the sample, the average CDRS-R score at baseline was 63.2 (*SD* = 14.9) and at follow-up it was 47.7 (*SD* = 17.3) (higher score indicate more severe depression). Table 4 shows the breakdown of individual participant raw CDRS-R scores. Within 8 weeks, scores changed in a positive direction for 18/20 participants. The scatterplot Figure 2 illustrates the distribution of the change in scores from baseline to follow-up where negative values indicate changes in a positive direction. When considering diagnostic cut-offs on the CDRS-R, 7/20 participants transitioned from diagnosable depression or early depression to no depression whereas the remaining 13/20 noted no change. The bubble-plot in Figure 2 illustrates the number of participants who changed clinical status (positive direction, no change), relative to their baseline and follow-up raw scores.

**Table 4:** CDRS-R raw scores at baseline (week 0) and follow-up (week 8).

ID	CDRS-R raw scores						
	Raw score		Diff.	Direction of change^a^ Weeks 8–0	Group^b^		Direction of change^c^ Weeks 8–0
	Week 8	Week 0			Week 8	Week 0	
01	49	47	−2	Negative	C	C	None
02	51	72	+21	Positive	C	C	None
03	29	38	+9	Positive	A	B	Positive
04	28	42	+14	Positive	A	C	Positive
05	40	54	+14	Positive	B	C	Positive
06	26	57	+31	Positive	A	C	Positive
07	21	63	+42	Positive	A	C	Positive
08	66	72	+6	Positive	C	C	None
09	46	62	+16	Positive	C	C	None
10	45	74	+29	Positive	C	C	None
11	85	90	+5	Positive	C	C	None
12	59	61	+2	Positive	C	C	None
13	51	47	−4	Negative	C	C	None
14	57	88	+31	Positive	C	C	None
15	53	69	+16	Positive	C	C	None
16	81	83	+−2	Positive	C	C	None
17	29	56	27	Positive	A	C	Positive
18	58	66	+8	Positive	C	C	None
19	41	47	+6	Positive	C	C	None
20	38	75	+37	Positive	B	C	Positive

^a^ Direction of change based on CDRS-R raw scores at week 8 compared to baseline (week 0) is indicted as positive if scores were higher at baseline (denoting more severe depression) than at 8 weeks.

^b^ Groups based on CDRS-R raw score thresholds: A ≤ 34 no depression, B = 35–40 emerging/early depression, C > 40 diagnosable depression.

^c^ Direction of change based on clinical status at week 8 compared to baseline (week 0) is indicated as “positive” if a participant changed groups in descending alphabetical order i.e., from C to B or A.

#### 3.5.3 Behavioural activation for depression scale- short form (BADS-SF)

The BADS-SF average score across all participants increased from 22.1 (*n* = 16, *SD* = 7.3) at baseline to 26.7 (*n* = 20, *SD* = 8.3) at 8-weeks follow-up, indicating increased behavioural activation and reduced avoidance in the sample overall. It is noteworthy that 4 participants completed the full version of the BADS (25 items) at baseline, which proved too cumbersome in combination with the other outcome measures; therefore, we replaced it with the short form (9 items) to reduce participant burden.

#### 3.5.4 Child health utility-9 dimensions (CHU-9D)

Most of the nine domains showed a positive direction of change, except for school and daily routine. The mean utility value was 0.718 (*SD*: 0.097) at baseline and 0.764 (*SD*: 0.108) at follow-up. The value range at baseline was 0.515–0.884 and at follow-up 0.507–0.915.

#### 3.5.5 Resource utilisation questionnaire for adolescents (RUQ-A)

Among primary and community-based healthcare and social services, only GP, practice nurse, social worker and online/telephone counselling were accessed by participants. For school-based services, alongside teachers, school nurses, and school counsellors, participants also reported talking to pastoral care leads, heads of year, and attendance officers about their wellbeing. Seven participants accessed child and adolescent mental health services at baseline and two did so at follow-up. Two participants used private counselling services at baseline, paying £12 and £40 respectively.

Secondary healthcare services such as hospitals were rarely used. Three participants had an outpatient appointment at baseline, whilst at follow-up two attended A&E and one had an outpatient appointment. None were admitted or received daycare. Two participants reported taking medication but only one of them was taking prescribed medicines.

Most participants took days off from school/work, 15 at baseline and 16 at follow-up; most were accompanied by a parent/guardian. The mean number of days absent was 10.8 days (*SD*: 7.7) over the 8 weeks before baseline and 7.0 days (*SD*: 7.3) over the 8 weeks after baseline. The maximum number of days absent was 24 days and 25 days respectively.

### 3.6 Intervention costs

Nine professionals attended the BA training with staff time costs estimated at £1,157 for the attendees and £510 for the two trainers. Three moderators addressed questions and comments during the first 3 weeks of the MOOC launch, costing £2,700 overall. The staff costs of the nine professionals completing the MOOC were £1,157, assuming all completed it according to schedule.

Supervision was held nine times; six were individual sessions and three were group sessions attended by two professionals. Most supervision sessions were conducted via MS Teams and lasted one hour, except for one lasting 30 min. The staff time costs of supervision were £447 for both professionals and the trainer. In total, training and supervision costs were estimated at £5,971, 65% of which were accounted for by the time spent on the MOOC.

In total, 140 sessions were delivered, associated with 172 h of staff working time. The mean total working time spent for the 19 participants who attended sessions was 9.1 h (*SD*: 2.2 h), ranging from 2 h to 13.5 h. The mean staff time costs of sessions were £207 (*SD*: £79) per participant.

### 3.7 Usual care survey

The usual care survey was open for completion between November 2021 and March 2022. Overall, 21 professionals responded to the questionnaire which was circulated to 40 services within the North-East of England. The services to which respondents belonged included voluntary/charitable organisations (*n* = 13), the NHS (*n* = 5), schools (*n* = 1), a charity partnered with the NHS (*n* = 1) and the local authority (*n* = 1). Respondents noted that, in their day-to-day practice, as little as 10% and as much as 80% of the young people whom they support would experience low mood. Where services had to signpost young people with low mood to alternative sources of support the reasons given for this were: having other organisational priorities, considering the depression too severe or complex, having a lack of appropriately trained staff and having limited staff availability.

Usual care took place over 1–10 sessions, usually between 30 min and 1 h, and included: general education, Interpersonal Therapy (IPT), Cognitive Behavioural Therapy (CBT), Behavioural Activation, counselling, pastoral support, psychotherapy, family therapy, emotional wellbeing support, art/drama/play/music therapy and youth work. Interventions were delivered face-to-face, by phone or online, both individually and in groups. Session frequency varied from weekly and monthly to ad-hoc/as needed. Staff were assistants and support workers (e.g., family support workers, teaching assistants, emotional literacy support workers), professionals trained in counselling, low intensity interventions (e.g., psychological wellbeing practitioners) and specialist high intensity interventions (e.g., CBT therapists, family therapists).

### 3.8 Stakeholder consultations

#### 3.8.1 Consultation round 1

Supplementary Table S2 summarises the key feedback and the actions taken during the first round of stakeholder consultations. In general, all groups agreed that the design and layout of the BA manual was clear, straightforward and should appeal across the target age range (12–18), sexes and gender identities. The content was also agreed overall to be understandable and appropriately worded. Some alterations however, regarding both the manual content and intervention structure were proposed. In several cases we directly adopted the recommendations, for instance introducing case study and role-playing examples as part of professional training. In other instances, we reviewed and refined the content or process in question or created something new inspired by the suggestion—the “activity bank” being one key example.

#### 3.8.2 Consultation round 2

Our stakeholder group made additional recommendations to refine the manual further (see Supplementary Table S3) which we incorporated. We took on board the stakeholders' suggestion to clarify medication details (if needed) after the young people complete the RUQ-A questionnaire. We also considered whether it would be better to refer to “mild-to-moderate depression” or to “low mood” in the BA manual, or indeed to use another term. We had encountered a variety of perspectives when speaking to schools and charities during recruitment to the feasibility study. Some felt the term “depression” was unjustified without a formal clinical diagnosis, as well as potentially unhelpful and 'stigmatising', while others considered that “low mood” trivialised what was being experienced. As a compromise, we continued to use “depression” as the default term in the manual but also stated, where the term is first introduced and described, that the young person may wish to use other terms e.g., “low mood” or “feeling down”.

### 3.9 Discussion

Increasing demand for mental health care, but limited staff capacity, have led to high entry thresholds for child and adolescent specialist services, which prioritise severe and complex clinical presentations; therefore, young people with mild-to-moderate problems, including depression, need to access help outside specialist services. This study assessed the feasibility of delivering BA within community settings to inform a large RCT that will test BA's clinical and cost-effectiveness. Two rounds of stakeholder consultations, as well as feedback from the professionals, researchers and young people who took part in the study, helped us refine the intervention materials, data collection tools and research processes.

Our excellent recruitment and retention rates showed that young people with mild-to-moderate depression are willing to take up and complete BA in a school, an NHS service and a charity, supported by professionals from different backgrounds and with different levels of expertise. This is in line with previous research in adult populations (7, 9) which have shown that BA can be supported by a diverse workforce, including more junior mental health workers. Furthermore, these studies have shown that junior mental health workers can achieve similar results at a lower cost than senior therapists (7, 9).

A feasibility study is not designed to test clinical effectiveness, but it can give a signal as to whether a follow-up RCT is worthwhile based on the direction of change from baseline to follow-up. Scores changed in a positive direction for around three-quarters of our participants across all outcome measures. When considering diagnostic cut-offs, the transition from clinical or sub-clinical to non-clinical status was more pronounced for the RCADS-25 than for the CDRS-R, and for depression than for anxiety.

It is noteworthy that “no change” in the clinical status for anxiety was observed among participants whose anxiety was within the “non-clinical” range at baseline, inferring a floor effect. It is unsurprising that the positive direction of change for anxiety was not as prominent as that for depression, given that our sample was recruited based on depression symptoms and the intervention itself was designed for depression. Still, we observed a positive direction of change for those participants whose anxiety scores were above the clinical threshold.

Overall, the data gathered in the RUQ-A was as we expected; healthcare and social service use mainly fell on school and primary care settings. This was anticipated in the development of the measure, as our study population—young people with mild-to-moderate depression—spend most of their time in school and their condition is not severe enough to typically warrant secondary care or medication. We have nevertheless added a question into the RUQ-A to explore medication use. In a future RCT, questions about medication use and secondary care access can be kept brief.

The survey findings demonstrated the variability in the types of support and the different levels of interventions offered to young people with depression; most importantly, the findings reassured us that BA was not widely used in community sites, therefore usual care was a suitable comparator for BA in a future RCT. The survey also showed the wide range of professionals responsible for providing support and interventions in different settings. These findings concur with previous studies in which pathways of mental health care for children and adolescents are loosely defined and inconsistent across different services and community settings (37–40). A future RCT needs to tailor training and supervision to the care pathways and professional skillset in each community setting, considering where our intervention aligns with existing practices and where therapists need to adapt to a new way of working.

#### 3.9.1 Strengths and limitations

Recruiting above our target indicated the willingness of young people and professionals to take up and use the intervention. Furthermore, for the 19 out of 20 young people who received BA, the mean number of sessions attended was 7.4 (*SD*: 1.5), suggesting that the intervention was deemed to be engaging. By recruiting young people from three types of setting—a school, a charity and a health service, we demonstrated that the intervention has ecological validity in its flexibility to be delivered in different community settings and by different professionals.

A battery of self-report questionnaires completed by the participants, a survey completed by professionals, and two series of stakeholder consultations with young people, parents, and professionals gave us robust information about the face validity of our intervention and research materials and processes, and enabled us to refine them in preparation for an RCT. Moreover, the survey reassured us about choosing usual care as the comparator.

Due to time constraints, we were only able to conduct a follow-up assessment at 8 weeks post-baseline whereas a future RCT will have a longer follow-up (at least 6 months). Also, four participants did not finish their treatment within 8 weeks, so their follow-up assessment was completed before their last BA session. Furthermore, with 17/20 participants identifying as female, the generalizability of the findings to a more gender-diverse population is limited. Still, the positive direction of change across all outcome measures justified the use of BA as an intervention that aims to improve depression in young people.

## 4 Conclusions

The objectives of this feasibility study were met in full by achieving excellent intervention uptake and adherence, retention to follow-up and data completeness. The direction of change from baseline to follow-up across multiple measures suggests that brief BA has the potential to reduce symptoms of depression and comorbid anxiety. A fully powered multi-site RCT is currently underway evaluating the clinical and cost-effectiveness of community-based BA against usual care.

## Data Availability

The raw data supporting the conclusions of this article will be made available by the authors, without undue reservation.
