# Supplementary material for: Community-based behavioural activation for depression in adolescents: feasibility study, survey and stakeholder consultations
Source: Front Child Adolesc Psychiatry. 2025 Jul 4;4:1596294. doi: 10.3389/frcha.2025.1596294 (PMC12271096; doi:10.3389/frcha.2025.1596294)
Supplement: Supplementary file 1 [file Datasheet1.docx]

Supplementary Material

# Tables

# Supplementary Table 1: Participant characteristics

| **ID** | **Gender** | **Age** | **Ethnicity** | **Religion** | **Education**  **Status** | **Employment**  **Status** | **Who living with** | **RCADS-25 Screening T score** |
| --- | --- | --- | --- | --- | --- | --- | --- | --- |
| 01/001 | Female | 13 | British | Protestant | Secondary | None | Parents | 66 |
| 01/002 | Female | 17 | British | Christian | Further/higher | Part-time | Parents | 73 |
| 01/003 | Female | 15 | British | Agnostic | Secondary | None | Parents | 65 |
| 01/004 | Female | 17 | British | Atheist | Secondary | None | Parents | 68 |
| 01/005 | Female | 14 | British | Catholic | Secondary | None | Parents | 75 |
| 01/006 | Female | 16 | British | Atheist | Further/higher | Part-time | Parents | 73 |
| 01/007 | Male | 17 | British | Atheist | Further/higher | Full-time | Parents | 65 |
| 01/008 | Female | 16 | British | Atheist | Secondary | Part-time | Parents | 79 |
| 01/009 | Female | 13 | British | Agnostic | Secondary | None | Parents | 71 |
| 01/010 | Female | 16 | British | Catholic | Further/higher | Part-time | Parents | 80 |
| 01/011 | Female | 14 | British | Atheist | Secondary | None | Parents | 80 |
| 02/001 | Female | 13 | White and Asian | Agnostic | Secondary | None | Parents | 80 |
| 02/002 | Male | 12 | Pakistani | Muslim | Secondary | None | Parents | 70 |
| 02/003 | Female | 14 | African | Protestant | Secondary | None | Parents | 68 |
| 02/004 | Female | 15 | Bangladeshi | Muslim | Secondary | None | Parents | 73 |
| 03/001 | Female | 18 | Indian | Hindu | Further/higher | None | Parents | 80 |
| 03/002 | Female | 15 | British | Catholic | Further/higher | None | Parents | 66 |
| 03/003 | Female | 14 | British | Agnostic | Secondary | None | Parents | 75 |
| 03/004 | Prefer not to say | 15 | British | Agnostic | Secondary | None | Parents | 66 |
| 03/005 | Female | 14 | British | Atheist | Secondary | None | Parents | 77 |

**Notes.** British: Denotes individuals identifying themselves as British, English, Welsh, Scottish or Northern Irish

**Supplementary Table 2:** Recommendations and responses from first stakeholder consultation

| **Stakeholder recommendations** | **Our response** |
| --- | --- |
| ***Manual*** | |
| More clearly link activities scheduled to weekly calendar to values | Adopted through ‘activities bank’ where activities are mapped to different ‘life pie’ areas |
| Consider separate manual for professionals which would include case studies and role-play examples | Not adopted: Agreed there should be no content or ‘agendas’ hidden from the participant. We produced several case studies and role plays for professional training. |
| Consider adding night-time slot to activity calendar | An evening rather than a night slot was added to encompass activities happening late-on but without normalising/encouraging night-time activity |
| Adapt barriers worksheet to include questions around personal experience of barriers and thoughts about what might help in overcoming these | Adopted |
| ***Intervention structure and delivery*** | |
| Hold follow-up session four weeks after end of intervention | We agreed that this was not practical in schools/ third sector context. The young people are given the skills and advice on what to do over the next 4 weeks. |
| Concern as to support ending abruptly as soon as young person completes intervention when may need further help. Suggest devising protocol for signposting young person to further support if needed at the end of the intervention. | We agreed that this was not practicable in schools/ third sector context, however, include setting-up realistic expectations with parents and young people and ‘4 steps to recovery’ to maintain progress/prevent relapse |
| Concerns with respect to recruiting individuals with appropriate role and motivation and sufficient availability to deliver intervention in schools. Create protocol for identification and assignment of professionals within schools to deliver intervention. | We agreed that rather than having a prescriptive protocol, training would be provided for a wide range of professional roles in schools and flexible supervision throughout offered. |
| Concerns about scheduling and locating intervention sessions within schools (e.g. students being obviously pulled out of class, being seen attending venue tied to mental health or behavioural issues). Suggestion to create protocol/guide for schools to use in determining best scheduling and location practices. Offer option of taking sessions after school. Offer possibility of sessions via videoconference, or even telephone, from home. | We agreed that sessions should be offered virtually. We would also advise the school to speak to each individual young person to try and find a time that suits them. In schools the appointments could be offered at different times each week to ensure that the young person does not miss the same lesson. |
| Provide case study and role-playing examples as part of professional training | Adopted |
| ***Other materials*** | |
| RUQ-A questionnaire - consider reducing technicality of language | Adopted where possible but in certain instances some specific technical language needed for sake of specificity and clarity. |
| Consent forms - opinion voiced by one parent that 12-13 years olds should be allowed opportunity to consent | Ethics Board already decided that 12–15-year-olds can give assent only. |

**Supplementary Table 3:** Recommendations and responses from the second stakeholder consultation

| **Stakeholder Recommendations** | **Our response** |
| --- | --- |
| Activities calendar: provide a key to three ‘PAC’ activity scoring dimensions and 1-10 rating scale; provide an opportunity to rate quality of sleep; add space to describe mood in a couple of words alongside ‘PAC’ score | The key was added but it was decided that inviting participants to rate quality of sleep and describe mood would be moving to beyond the intervention’s focus on the core principles and practices of behavioural activation |
| Life pie: some suggested wording revisions and the inclusion into one of the pie segments of religious and other causes/beliefs plus adding pets to the ‘my people’ section | We adopted most of these very helpful suggestions. |
| Activity bank: there were concerns that the template was too complicated and crowded with not enough space to add in the activities | It was challenging to know how to address these concerns and still preserve all the core elements of the activity bank. We however moved the list of steps to achieving each goal into a separate worksheet. The groups’ concerns might have been allayed if it had been made clearer at the time that the young person would not be expected to complete the sheet on their own. |
| Four steps to recovery: several proposals were made for adding some illustrations/icons around the text. These included words/symbols of encouragement to the young person, pictures of sample activities that they might find helpful, and aide-memoire thumbnails of the various BA component exercises and associated templates. | We agreed that the aide-memoire thumbnails would be the most appropriate and useful. We were reluctant to include illustrations of any particular activities as what is beneficial for one might not be for another and also wish to encourage the young person to look back at their activity bank. |
